# Endoscopic ultrasound-guided hepaticogastrostomy with antegrade and bridging stenting for diffusely extending cholangiocarcinoma

**DOI:** 10.1055/a-2840-7847

**Published:** 2026-04-20

**Authors:** Takao Nishikawa, Shinji Morii

**Affiliations:** 1 13626 Department of Gastroenterology, Matsudo City General Hospital, Chiba, Japan

**Fig. 1:**
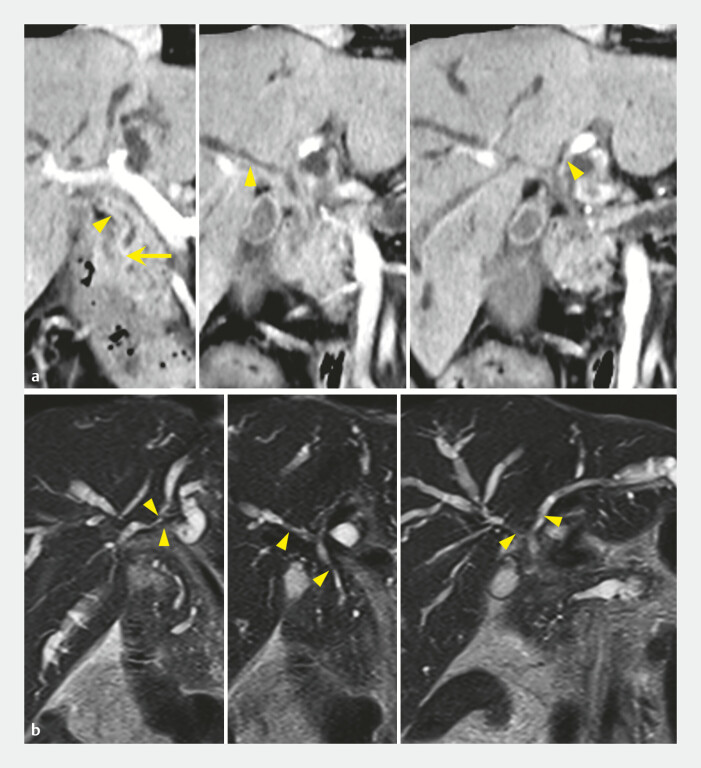
**a** Enhanced computed tomography showing distal bile duct obstruction caused by cholangiocarcinoma (arrow) and subsequent thickening of the bile duct wall extending bilaterally to the secondary bile duct branches (arrow heads). **b** Magnetic resonance cholangiopancreatography showing bilateral narrowing of the bile ducts up to their secondary branches (arrow heads) and dilation of peripheral intrahepatic biliary branches.

**Fig. 2:**
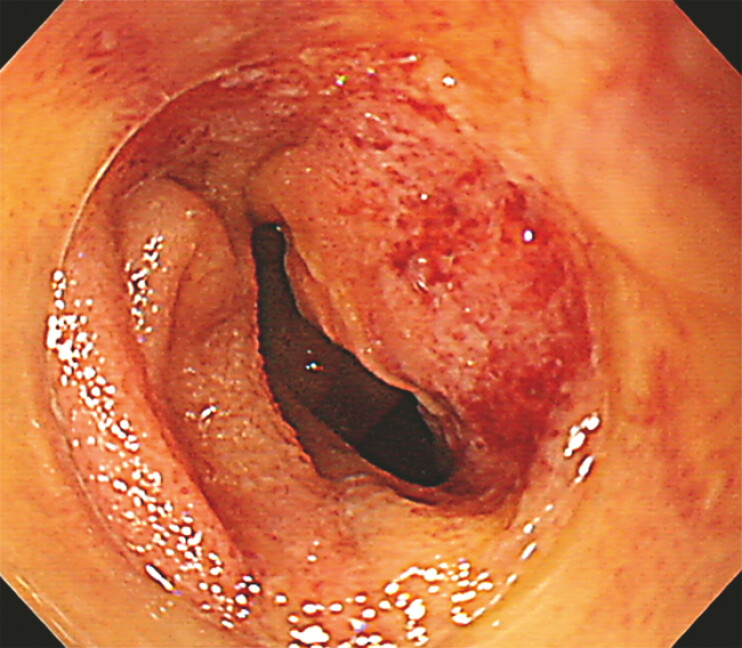
An endoscopic view showing tumor infiltration into the descending part of duodenum.

**Fig. 3:**
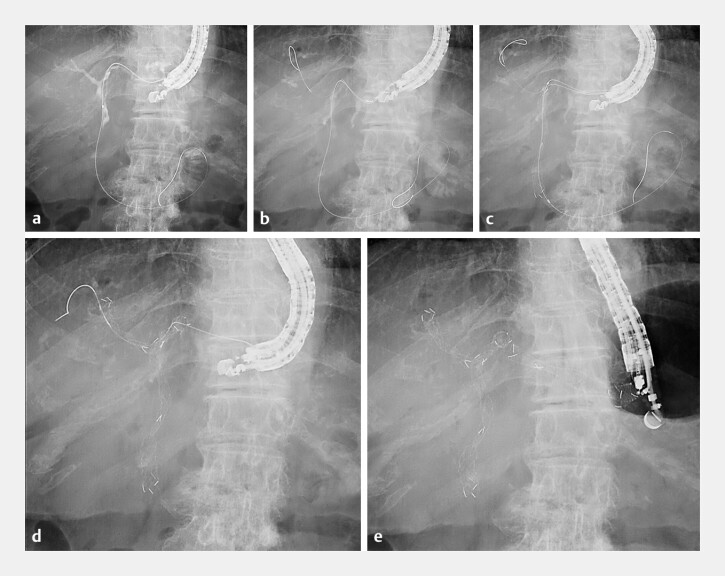
Fluoroscopic images of endoscopic ultrasound-guided hepaticogastrostomy. **a** The insertion of a guidewire through the major papilla into the duodenum via the hepaticogastrostomy route. **b** Another guidewire inserted into the right anterior biliary branch. **c** An uncovered self-expandable metal stent (SEMS) deployed from the common bile duct to the left hepatic duct via the major papilla. **d** Another uncovered SEMS deployed from the right anterior biliary branch to the left hepatic duct using the partial stent-in-stent method. **e** A partially covered SEMS deployed from the B3 branch of the left intrahepatic bile duct into the hepaticogastrostomy fistula. SEMS, self-expandable metal stent.

**Fig. 4:**
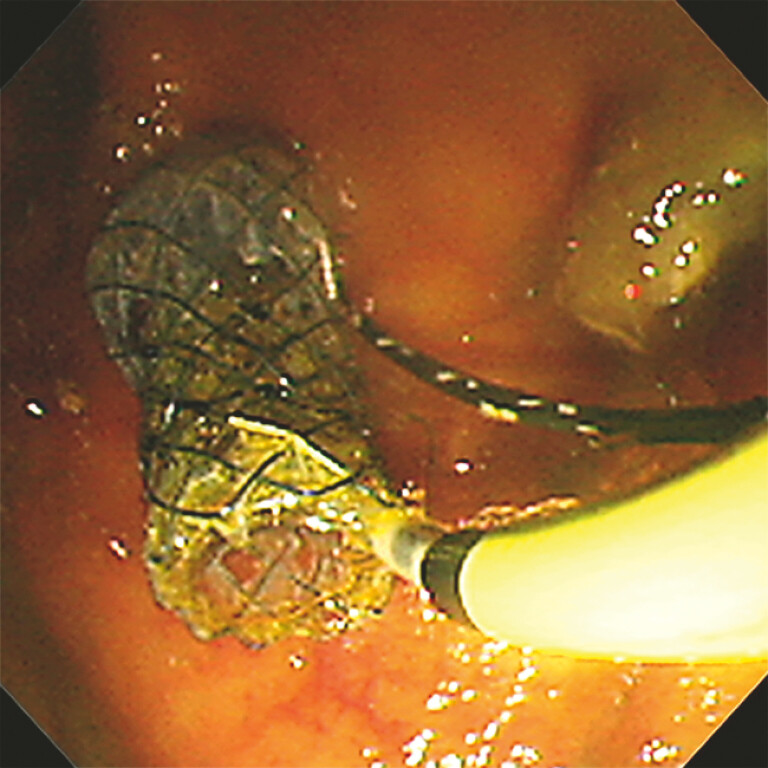
An endoscopic view showing a partially covered SEMS deployed into the hepaticogastrostomy fistula. SEMS, self-expandable metal stent.

An 81-year-old female patient with jaundice and a total bilirubin level of 25 mg/dL was diagnosed with distal cholangiocarcinoma extending bilaterally to the intrahepatic bile ducts ( Fig. 1 ). Although endoscopic retrograde cholangiopancreatography was attempted, the major papilla could not be approached due to tumor infiltration of the duodenum ( Fig. 2 ). Thus, endoscopic ultrasound-guided hepaticogastrostomy (EUS-HGS) was performed to achieve biliary drainage ( Video 1 ), where the B3 branch of the left intrahepatic bile duct was punctured from the stomach ( Fig. 3 **a** and **b** ). For antegrade stenting, an uncovered self-expandable metal stent (SEMS) was deployed via the major papilla from the common bile duct to the left hepatic duct ( Fig. 3 **c** ). Next, using the partial stent-in-stent method, another uncovered SEMS was deployed from the right anterior biliary branch to the left hepatic duct to bridge bilateral biliary branches ( Fig. 3 **d** ). Finally, a partially covered SEMS was deployed from the B3 branch of the left intrahepatic bile duct into the hepaticogastrostomy fistula to sustain a re-intervention route in the case of stent failure ( Fig. 3 **e** and Fig. 4 ). No procedure-related adverse events occurred. Within 3 days, the total bilirubin level decreased to less than one-third the initial measurement.

Endoscopic ultrasound-guided hepaticogastrostomy with antegrade and bridging stenting was performed to achieve biliary drainage in a patient with diffusely extending cholangiocarcinoma that had infiltrated the duodenum.Video 1

**Fig. 5:**
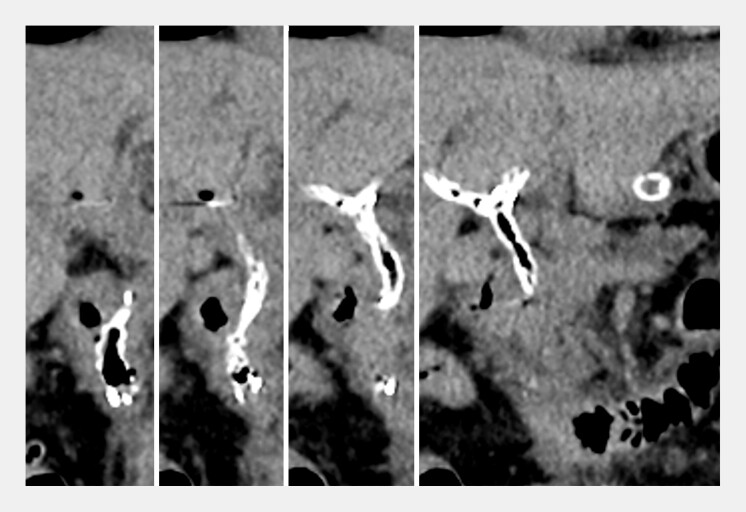
Computed tomography showing three SEMSs used in antegrade and bridging stenting, along with hepaticogastrostomy. SEMSs, self-expandable metal stents.

When the transpapillary approach to the malignant biliary stricture is challenging, endoscopic ultrasound-guided biliary drainage is preferred over a percutaneous transhepatic biliary drainage because of better clinical efficacy and non-invasiveness 1 2 .The time to recurrent biliary obstruction is longer in EUS-HGS with antegrade stenting for malignant distal biliary obstruction than that without it 3 4 .Furthermore, for malignant perihilar biliary strictures, bilateral stenting results in longer stent patency than unilateral stenting 5 . In this case, where the stricture extended to bilateral biliary branches, additional bridging stenting resulted in successful biliary drainage ( Fig. 5 ). Multiple biliary stenting via EUS-HGS can be effective and safe for diffuse bile duct strictures.

Endoscopy_UCTN_Code_TTT_1AS_2AD
